# Norwegian Adolescents’ Multidimensional Understandings of Wellbeing: A Qualitative Study

**DOI:** 10.3390/bs16010081

**Published:** 2026-01-07

**Authors:** Sultana Ali Norozi, Anne Torhild Klomsten

**Affiliations:** Department of Education and Lifelong Learning (IPL), Norwegian University of Science and Technology (NTNU), 7491 Trondheim, Norway

**Keywords:** wellbeing, adolescents, perceptions, multidimensional, physical wellbeing, mental wellbeing, social wellbeing, strategies to improve wellbeing

## Abstract

Adolescence is a period of significant physical, cognitive, and social change, during which various challenges can affect wellbeing. Understanding adolescents’ own perceptions of wellbeing is crucial for developing effective support strategies. This study explores how Norwegian adolescents conceptualize their wellbeing. Using a qualitative design, semi-structured interviews were conducted with 26 adolescents aged 16–19 years, and the data are analyzed thematically. The findings reveal that wellbeing is viewed as multidimensional, encompassing physical health, social relationships, academic performance, and personal growth. Family support and community engagement emerge as central to promoting adolescents’ wellbeing. The study provides valuable insight into how young people in Norway define and experience wellbeing, emphasizing the need to integrate their perspectives into policies and interventions aimed at supporting adolescent development and mental health.

## 1. Introduction

Wellbeing is a fundamental aspect of human life and a key determinant of individuals’ quality of life. The World Health Organization defines health as a state of complete physical, mental, and social wellbeing, rather than merely the absence of disease or infirmity (WHO, 2018). Adolescence represents a critical developmental period marked by the transition from childhood to adulthood, during which young people experience profound physical, psychological, emotional, and social changes. These changes can significantly influence adolescents’ wellbeing, making this life stage particularly sensitive to both protective and risk factors. Promoting wellbeing during adolescence is therefore a central concern for individuals, educational institutions, and society at large.

In recent years, increasing attention has been directed toward adolescents’ wellbeing by a wide range of stakeholders, including parents, teachers, policymakers, researchers, and organizations involved in young people’s leisure activities. Despite this growing emphasis, wellbeing remains a concept that is variably defined and theorized across disciplines (Diener, 1984; Ryff, 1989; Hooker et al., 2020; Norozi, 2023). At the same time, adolescents’ wellbeing is shaped by multiple interrelated factors, such as physical health, social relationships, academic demands, personal growth, and the broader community environment. Research indicates that adolescents who perceive their wellbeing positively tend to demonstrate better mental health outcomes, lower engagement in risky behaviors, and higher academic performance (Eckersley et al., 2006; J. White, 2011).

Norway provides a particularly relevant context for examining adolescent wellbeing due to its high standard of living and strong policy commitment to health and education. In August 2020, a comprehensive revision of the national curriculum for primary, lower, and upper secondary education was introduced across the Norwegian K–12 system. A central feature of this reform was the inclusion of the interdisciplinary theme Public Health and Life Skills, which is integrated across all subjects. This theme aims to promote students’ physical and mental wellbeing, strengthen their capacity to manage social and academic challenges, and support the development of positive relationships and emotional competence. Despite this strong policy emphasis, there remains limited empirical research on how Norwegian adolescents themselves understand and experience wellbeing (Ekornes & Øye, 2022). Adolescents’ perspectives are particularly important, as their understandings of wellbeing are shaped by their developmental stage, lived experiences, and social contexts, and may differ from adult or policy-driven conceptualizations. Exploring adolescents’ own perceptions can therefore provide valuable insight into the factors that support or hinder their wellbeing, as well as the challenges they encounter in striving for a good life.

Understanding young people’s perspectives on wellbeing is also essential for informing policies and interventions aimed at supporting present and future generations. Adolescents represent a key group for promoting positive health behaviors and preventing adverse outcomes, including mental health difficulties and chronic health conditions. Against this backdrop, the present study aims to explore Norwegian adolescents’ perceptions of wellbeing, the factors they identify as contributing to their wellbeing, and their lived experiences of wellbeing. By foregrounding adolescents’ voices, this study seeks to contribute to a deeper and more contextually grounded understanding of wellbeing and to inform efforts to promote young people’s health and development, particularly within educational settings where they spend a significant part of their daily lives.

### 1.1. Multifaceted Nature of Wellbeing

There exists notable international and national concern regarding the promotion of wellbeing within educational institutions, albeit with variations in the operationalization of the term based on the specific objectives, temporal context, and geographical location of the research. Traditionally, wellbeing was associated with notions such as welfare, utility, and happiness (Thorburn, 2018). However, in contemporary discourse, an expanded and diverse array of themes (Roscoe, 2009; Linton et al., 2016; Hooker et al., 2020; Montoya & Summers, 2021), often used interchangeably, are encompassed within the concept of wellbeing, including the emergent discourse of sustainability (Spratt, 2017). Wellbeing in education is a holistic concept that encompasses physical, mental, social, psychological, emotional, cultural, and environmental aspects (Norozi, 2023). It is not only about academic achievement, but also about the social, cultural, emotional, psychological, environmental, and physical development of the student. Students who are happy, healthy, and feel safe in school environments are more likely to be successful in their academic pursuits. The concept of wellbeing is a subject of extensive discussion across various disciplines; however, a precise definition remains elusive, as indicated by Hooker et al. (2020). Scholars and practitioners worldwide have dedicated efforts to comprehend and promote wellbeing from diverse perspectives. Consequently, several common threads in understanding wellbeing have emerged, transcending cultural and societal disparities. One pivotal thread in the understanding of wellbeing is its recognition as a multidimensional construct. While the number of dimensions ascribed to wellbeing ranges from three to twelve (Roscoe, 2009), there is general consensus among scholars that it is a multidimensional concept. Notably, the World Health Organization (WHO) definition of wellbeing is widely accepted, emphasizing that it extends beyond the mere absence of illness and infirmity. These dimensions of wellbeing are interconnected and function synergistically, with each dimension being integral to the overall state of wellbeing. The predominant dimensions frequently explored in prominent theories encompass social, physical, psychological, intellectual, spiritual, emotional, environmental, cultural, and financial aspects (Roscoe, 2009; Adams et al., 2000; Linton et al., 2016; Hooker et al., 2020; Montoya & Summers, 2021; Norozi, 2023).

Wellbeing has become an increasingly important issue in international education policy-making in recent years. With the recognition that wellbeing is a fundamental element of human development and is a key factor in promoting successful education, many international organizations and governments have prioritized wellbeing in their policy agendas. The World Health Organization’s (WHO) Health Promoting Schools framework provides a comprehensive framework for promoting health and wellbeing in schools, based on the recognition that schools play a critical role in supporting the physical, mental, and social health of students. The Health Promoting Schools framework includes several key components, for example, creating a supportive school environment, strengthening health education, providing opportunities for physical activity, and addressing health and social inequities. The Health Promoting Schools framework has been implemented in many countries around the world and has been shown to be effective in promoting students’ health and wellbeing. The wellbeing and health behavior of young people in 50 countries have been under investigation since 1982 by the collaborative efforts of the Health Behavior in School-Aged Children (HBSC) and the WHO Regional Office for Europe. These studies yield valuable insights that contribute to the decision-making processes of governing bodies and aim to enhance the lives of young individuals (Inchley et al., 2020). Recent investigations conducted by the HBSC demonstrate a decline in life satisfaction among young people in the majority of Nordic countries (Inchley et al., 2020). Furthermore, the global COVID-19 pandemic has exacerbated the negative impact on the quality of life experienced by young individuals (Haugseth & Smeplass, 2021). Recognizing the importance of wellbeing in education, the Organization for Economic Cooperation and Development (OECD, 2021) has initiated the Study on Social and Emotional Skills (SSES) to evaluate and gauge wellbeing. The SSES survey represents a paradigm shift, shifting the focus to non-cognitive facets of learning. This program places particular emphasis on the psychometric science of personality measurement, which encompasses five overarching domains known as the “Big Five” model.

In Norway, there are several policy documents related to wellbeing in school. The Norwegian Education Act (1998) is the overarching law that governs education in Norway. It states that education should be based on fundamental values such as respect for human rights, democracy, and sustainable development. The act also emphasizes the importance of providing students with a safe and inclusive learning environment (The Norwegian Education Act, 1998, § 9 A-2). The National Curriculum (National Curriculum for Knowledge Promotion in Primary and Secondary Education and Training, LK20) is a framework that sets out the goals and objectives for primary and secondary education in Norway. The curriculum emphasizes the importance of promoting students’ physical, mental, and social wellbeing, as well as their academic development. It also emphasizes the need to provide students with a broad and balanced education that prepares them for lifelong learning. The Action Plan for the Promotion of Mental Health is a national strategy that aims to promote mental health and prevent mental illness among children and adolescents in Norway. The plan includes several initiatives aimed at promoting mental health in schools, such as providing training for teachers and school staff and promoting student participation in school decision-making.

Overall, these policy documents reflect a strong commitment in Norway to promoting young people or adolescents’ wellbeing in school, with an emphasis on providing a safe and inclusive learning environment and promoting health and wellbeing.

### 1.2. Earlier Research About Adolescents’ Perceptions of Wellbeing

Research on adolescents’ wellbeing in education has been ongoing for several decades, with a particular focus on the impact of school environments and educational policies on students’ wellbeing (Pulimeno et al., 2020). In the era of 1980s, researchers began to explore the concept of subjective wellbeing, which refers to individuals’ evaluations of their own lives and their emotional experiences. Studies in this area focused on how young people’s subjective wellbeing is related to factors such as family relationships, social support, and academic achievement (Diener, 1984; Ryff, 1989). In the late 1990s and early 2000s, researchers began to adopt a more holistic approach to understanding young people’s wellbeing, recognizing that it encompasses physical, emotional, and social dimensions. Studies (Headey, 1998; Sagiv & Schwartz, 2000; Triandis, 2000) in this area focused on how young people’s overall wellbeing is related to factors such as health behaviors, physical activity, social support, and engagement in school. As Diener and Eunkook (2000) state, wellbeing “can represent the degree to which people in each society are accomplishing the values they hold dear” (p. 4). There has been increasing interest in understanding how young people’s perceptions of wellbeing may be influenced by broader societal factors, such as economic inequality, social media use, and political instability (R. White & Wyn, 2004; Eckersley et al., 2006; Easthope & White, 2006; Ecclestone & Hayes, 2009; Watson et al., 2012). Overall, research on adolescents’ or young people’s perceptions of wellbeing has evolved over time, reflecting changes in our understanding of what constitutes wellbeing and how it is influenced by various factors. While early studies focused on individual-level factors, more recent studies have adopted a more contextual approach, recognizing that young people’s wellbeing is shaped by a range of social, economic, and political factors. Exclusive emphasis on discrete factors that impact individual wellbeing fails to acknowledge the crucial role that social processes play in shaping overall wellbeing. To illustrate, the condition of homelessness emerges as a consequence of one or more social dynamics, such as an insecure residential setting (R. White & Wyn, 2004). Within the realm of youth research, the notion of wellbeing encompasses not only the resultant outcomes and emotional states but also recognizes the intricacies of social processes that profoundly shape the lives of young individuals (R. White & Wyn, 2004). Nevertheless, despite the adoption of the wellbeing concept by youth researchers to explore the experiences and social dynamics of young people, a uniform definition remains elusive, and theoretical frameworks are still evolving (Eckersley et al., 2006). “The very distinction between mind and body, between mental, physical, and social elements of life for young people may be part of the problem, because they mask the real, complex struggles that young people have in making a life” (R. White & Wyn, 2004) (p. 215).

Taken together, existing research demonstrates that adolescent wellbeing is a complex and multifaceted phenomenon influenced by individual experiences as well as broader social, cultural, and structural conditions. While previous studies have provided important insights into factors associated with young people’s wellbeing, such as family relationships, school engagement, and social support, much of this research has relied on adult-defined conceptual frameworks or quantitative indicators. As a result, adolescents’ own understandings of wellbeing and their lived experiences remain underrepresented, particularly in qualitative research that allows young people to articulate what wellbeing means to them in their own words. Furthermore, despite the growing international focus on wellbeing in education and the strong policy emphasis on wellbeing within the Norwegian context, there is limited empirical research examining how Norwegian adolescents perceive wellbeing and which factors they themselves identify as contributing to their wellbeing and everyday experiences. Given the context-dependent and subjective nature of wellbeing and the central role of schools in adolescents’ lives, there is a clear need for research that foregrounds young people’s perspectives within specific national and educational settings. Responding to this gap, the present study explores Norwegian adolescents’ perceptions of wellbeing and examines the key factors they identify as contributing to their wellbeing and experiences of wellbeing. By centering adolescents’ voices, this study seeks to provide a contextually grounded understanding of wellbeing that can inform educational practice, policy development, and future research.

### 1.3. Research Questions

In light of this context, the present paper delves into the ensuing inquiries:

RQ1. What are the perceptions of wellbeing among Norwegian adolescents?
RQ2. What are the key factors that contribute to their wellbeing and their experiences of wellbeing?

## 2. Method

The study employed a qualitative research design, and data was collected between April and December 2019. Participants were adolescents aged 16–19 years who were enrolled in upper secondary school in Norway and who voluntarily agreed to take part in the study. The primary inclusion criteria were age (16–19 years) and current enrolment in upper secondary education; no additional exclusion criteria were applied. Informed consent was obtained from both the participants and their parents/guardians. The sample comprised 9 male and 17 female participants. The interviews were conducted using a semi-structured format, and the participants were asked open-ended questions about their perception of wellbeing, the factors that contribute to their wellbeing, and their experiences of wellbeing. The interviews were conducted in a quiet and private room in the school, and each interview lasted between 45 and 60 min. The participants were encouraged to share their experiences, thoughts, stories, and feelings regarding the topic of the study. The interviews were audio-recorded and transcribed verbatim. The data were analyzed using thematic analysis. The transcripts were read several times to gain a holistic understanding of the data. The initial codes were generated, and the codes were grouped into sub-themes based on their similarities and differences. The sub-themes were reviewed and refined, and the final themes were identified. To enhance analytic rigor, the analysis followed an inductive and iterative process, with repeated engagement with the data and ongoing reflective discussions between the authors to critically examine interpretations and ensure that themes were grounded in participants’ accounts rather than in pre-existing assumptions.

One of the strengths of this study is its focus on adolescents’ perspectives on wellbeing, which is a relatively under-researched area. Another strength of the study is its use of a qualitative approach, which allowed for a rich and detailed exploration of adolescents’ perceptions of wellbeing. However, there are also some limitations to the study that should be noted. The study sample was relatively small, and the findings may not be representative of all Norwegian adolescents’ perceptions of wellbeing. In addition, the study was conducted in a specific geographic location and cultural context, which may limit the generalizability of the findings to other contexts. Another substantial point is that all adolescents may not have the necessary self-awareness or communication skills to accurately express their perceptions of wellbeing and this may affect the validity of the data collected. Despite these limitations, the study provides valuable insights into how young people understand and experience wellbeing. Overall, this study makes a valuable contribution to the literature on adolescents’ perceptions of wellbeing and provides useful insights for those working with young people to promote their development and wellbeing.

## 3. Results

Data reveals that adolescents have unique perspectives on wellbeing that are diverse and complex, reflecting their unique experiences and contexts. There are some common understandings that are shaped by their age, experiences, and the context in which they live.

The analysis of the data unveiled six primary themes, accompanied by several sub-themes, which reflected the adolescents’ perspectives on wellbeing. The main themes and sub-themes are presented below in Table 1.

### 3.1. Decoding Wellbeing in Adolescence: Subjective and Objective Dimensions

When asked what wellbeing meant to them, most of the adolescents described wellbeing as a complex and multidimensional concept rather than a single, clearly defined state. Participants emphasized that wellbeing involved multiple interrelated aspects of life, including physical, mental, emotional, and social elements.

One first-year female student explained: “For me, wellbeing is all about feeling good physically, mentally, socially and emotionally. It’s about taking care of yourself but also making time for friends and family and doing things that make you happy.” Similarly, a second-year male student described wellbeing in terms of acceptance and self-expression: “Wellbeing is about being able to express yourself and be who you are without fear of judgement or discrimination. It’s about feeling accepted and valued for who you are.”

Across participants’ accounts, wellbeing was described both as a subjective experience, such as feeling happy, satisfied, or content, and as something connected to objective life conditions, including physical health, access to support, and opportunities in daily life. Some adolescents emphasized emotional balance and the ability to cope with challenges, rather than the absence of difficulties. A first-year female student stated: “To me, wellbeing is about being able to cope with the ups and downs of life. It’s about having the resilience to bounce back from challenges and not letting them get the best of you.” Overall, participants’ descriptions highlighted wellbeing as a dynamic state that involved both internal experiences and external conditions, with individual interpretations shaped by personal circumstances.

### 3.2. Physical Health: The Connection Between Lifestyle Factors and Wellbeing

Physical health was frequently described as an important component of overall wellbeing. Participants spoke about the importance of healthy eating, regular physical activity, and sufficient sleep. Many adolescents expressed a desire to maintain healthy routines, while also describing challenges in doing so consistently. A third-year female student reflected on competing demands: “I know that eating healthy, exercising regularly, and getting enough sleep are all important, but it is really hard to make time for all of them. Between school, activities, and socializing, there just aren’t enough hours in the day sometimes.”

Several participants linked their physical wellbeing to daily routines and time management. Sleep, in particular, was mentioned as difficult to prioritize. A first-year male student explained: “I know that I feel better when I get enough sleep, but it’s hard to prioritize it when there’s always something going on. Sometimes I have to stay up late to study or hang out with friends.” Feelings of frustration were common when adolescents felt unable to balance school demands, social life, and self-care. A first-year female student stated: “It’s frustrating… I want to take care of myself, but it feels like there’s not enough time in the day. I wish there were more hours so I could fit everything in.” These accounts illustrate how adolescents experienced physical wellbeing as closely connected to everyday pressures and competing responsibilities.

### 3.3. The Role of Social Relationships in Wellbeing

Social relationships emerged as a central theme in adolescents’ accounts of wellbeing. Participants described a range of relationships that they associated with feeling well, including relationships with family members, friends, romantic partners, and broader social networks. Sub-themes within this category included feelings of belonging, trust, care, respect, love, and having a secure and supportive home environment. Social media was also frequently mentioned as part of adolescents’ social lives. Family relationships were commonly described as a primary source of support. A second-year female student stated, “My family is really important to me. They’re always there for me and they support me no matter what.” Many participants emphasized the emotional security they experienced through close family ties. Friendships were highlighted as particularly important for everyday wellbeing. One second-year student explained, “Friends are everything. They’re the ones who make life fun and interesting. Having close friendships is really important to me. I feel like I can be myself around my friends and they accept me for who I am.”

Some adolescents also reflected on changes in friendships over time. A first-year male student noted, “It’s hard when friendships change or drift apart, but I think it’s a natural part of growing up. You learn who your real friends are and who you can count on.” Participants expressed differing views regarding romantic relationships. Some described romantic relationships as meaningful and supportive, while others indicated that they were not a current priority. A third-year male student shared, “Being in a relationship can be complicated… but when it’s good, it’s really good. It’s nice to have someone to share things with and support you.” In contrast, a first-year female student stated, “I’m not really interested in dating right now. I’d rather focus on my friendships and my own personal growth.”

Social relationships were also discussed in relation to digital interaction. Adolescents described social media as an important space for connection, while also noting challenges associated with its use. One participant explained, “I feel like there’s a lot of pressure to look a certain way, especially on social media. It’s hard not to get caught up in it and start to feel like you’re not good enough.” These accounts indicate that online interactions formed part of adolescents’ broader social experiences and influenced how they perceived their wellbeing.

### 3.4. The Dynamics of Mental Health

Mental health was frequently described by participants as closely connected to their overall wellbeing. Several adolescents spoke about mental health and wellbeing as overlapping or interchangeable concepts. A third-year student explained: “For me, mental health is just as important as physical health. If I’m not feeling good mentally, it affects everything else in my life. That’s why I try to take care of myself in all areas … and do things I enjoy. But I also know that it’s okay to not be okay sometimes.”

Participants described a range of actions they associated with taking care of their mental health. These included doing activities they enjoy, practicing mindfulness or meditation, setting boundaries with social media, and seeking professional help. However, many reported that they did not engage in these practices regularly. Lack of time was frequently mentioned as a barrier. One participant stated: “It’s not just about when you’re going through a hard time, it’s also about taking care of yourself always. That might mean practicing mindfulness, doing things that make you happy, or setting boundaries with social media etc. … but it is hard to remember these always and when you don’t have enough time.”

Several adolescents mentioned spending time in nature as something they experienced as beneficial for their mental health. Participants referred to outdoor activities such as walking, hiking, or spending time in green spaces. Some adolescents used the Norwegian expression “ut på tur, aldri sur [out on a tour, never grumpy]” when describing these experiences, linking outdoor activities to improved mood and emotional balance.

When discussing help-seeking for mental health concerns, participants commonly referred to stigma. Although many stated that they personally did not view mental health difficulties as stigmatizing, they expressed discomfort about disclosing such challenges within their social circles. Several participants said they would hesitate to talk openly about mental health issues if they needed support. Many adolescents also expressed a desire to learn how to handle difficult and demanding life situations while maintaining their wellbeing. Participants spoke about the importance of being able to cope with challenges and remain positive. At the same time, they described uncertainty about how to develop these abilities or apply them in everyday situations when facing difficulties.

### 3.5. Academic Performance Pressure and Its Implications on Wellbeing

Academic performance pressure was frequently mentioned by participants as a factor that influenced their wellbeing. Adolescents described experiencing pressure related to grades, expectations, and concerns about their future, particularly during the later years of upper secondary school.

A third-year female student described this pressure as follows: “I feel like there’s so much pressure to perform academically, especially as you get closer to the end of high school. You constantly start feeling that your grades will determine your future, and that can be really overwhelming. I know that I personally struggle with anxiety when it comes to school, and it can be hard to find a balance between doing well and taking care of myself.”

Participants spoke about how academic demands affected their emotional state and ability to maintain wellbeing. Several adolescents described feeling stressed or anxious when trying to meet academic expectations while also managing other aspects of their lives. The school environment was described as an important factor in how adolescents experienced academic pressure. Participants reported that feeling supported by teachers and experiencing a safe school environment helped reduce stress related to school performance. One participant explained: “A supportive and safe school environment, particularly through the care and support of teachers, plays a vital role in alleviating academic pressure for me. When teachers demonstrate genuine care and offer support, it creates a nurturing atmosphere where I feel valued, understood, and motivated to succeed.”

Participants also described how teacher support and a positive school atmosphere encouraged them to seek help when needed and feel more confident in managing academic demands. Adolescents indicated that feeling cared for and supported at school influenced both their academic experiences and their overall wellbeing.

### 3.6. Personal Growth

Personal growth was described by participants as an important part of their wellbeing. Adolescents spoke about personal growth in relation to understanding themselves better, developing confidence, managing everyday challenges, and working toward personal goals. Participants referred to aspects such as self-awareness, self-discipline, autonomy, creativity, and finding meaning in life. Several participants described engaging in activities they associated with personal growth, including hobbies, sports, creative activities, and involvement in their communities. These activities were described as ways to express themselves, explore interests, and pursue goals. Adolescents also spoke about valuing opportunities to make a positive contribution to others or to work toward meaningful aspirations.

Participants described using different strategies to support their wellbeing and personal development. These included physical activity, mindfulness, talking to friends or family members, and engaging in hobbies. Some participants also mentioned seeking professional support when needed. A first-year female student described the importance of self-awareness in personal growth: “You need to know what makes you happy, what stresses you out, and what you’re capable of in order to make good changes… but it can be hard to know where to start, especially if you don’t have a lot of support. I wish there was some kind of platform that could help me to develop this.”

Many adolescents expressed a desire to learn how to handle difficult and demanding life situations. Participants referred to challenges such as academic pressure, conflicts with family and friends, social media, and uncertainty about the future. They described wanting tools and skills that could help them feel more in control of their lives and better able to manage these situations. One participant explained how engaging in activities helped them cope, while also highlighting the need for additional support: “I find that engaging in sports or hobbies is a great way to boost my mood and relieve stress. It gives me a sense of accomplishment and helps me forget about my problems for a while. But sometimes, life throws really hard situations at you, and it can be hard to know how to cope. I think it would be really helpful to have access to trained professionals who could help me develop the skills I need to handle those situations in a healthy way.” Participants also spoke about preferring opportunities where they could actively practice skills in supportive environments. Some adolescents mentioned interest in structured support, including guidance from teachers or other trusted adults, as well as the use of digital tools such as apps or online programs to support learning and coping.

## 4. Discussion

This study explored Norwegian adolescents’ perceptions of wellbeing (RQ1) and the factors they identify as contributing to their wellbeing and lived experiences (RQ2). By foregrounding adolescents’ own voices, the findings provide insight into how young people understand wellbeing within their everyday social, educational, and cultural contexts. The discussion below integrates these findings with existing research to deepen understanding of adolescent wellbeing in Norway.

### 4.1. Research Question 1: How Do Norwegian Adolescents Perceive Wellbeing?

The findings related to RQ1 demonstrate that Norwegian adolescents perceive wellbeing as a complex and multidimensional phenomenon. Adolescents described wellbeing as encompassing both subjective experiences such as feeling happy, balanced, accepted, and able to cope and objective conditions, including physical health, supportive relationships, and everyday resources. This aligns with longstanding theoretical and empirical work that conceptualizes wellbeing as more than the absence of ill health, emphasizing its multidimensional and relational nature (Diener, 1984; Ryff, 1989; Roscoe, 2009; Hooker et al., 2020; Norozi, 2023). Consistent with previous qualitative research on young people’s understandings of wellbeing (R. White & Wyn, 2004; Eckersley et al., 2006; Easthope & White, 2006; Bourke & Geldens, 2007a, 2007b; Fattore et al., 2009; Thorburn, 2018; Ekornes & Øye, 2022), adolescents in this study articulated wellbeing in diverse yet overlapping ways. Some emphasized internal emotional states and personal coping, while others highlighted external life conditions and relational support. This variation reflects the context-dependent nature of wellbeing and suggests that adolescents draw on both personal experiences and social environments when forming their understandings. Notably, several adolescents appeared to frame wellbeing primarily in terms of individual responsibility and self-management, with limited reference to broader structural or systemic factors. Similar patterns have been identified in earlier research, where young people tend to accept existing social and institutional arrangements rather than critically engaging with them (J. White, 2011; Thorburn, 2018). This finding has important implications for how wellbeing is addressed in educational and policy contexts, as it suggests that adolescents may benefit from greater support in recognizing and navigating the broader conditions that shape their wellbeing.

### 4.2. Research Question 2: What Factors Do Adolescents Identify as Contributing to Their Wellbeing and Lived Experiences?

In relation to RQ2, the findings highlight several interconnected factors that adolescents perceive as shaping their wellbeing, including physical health, social relationships, mental health, academic pressure, and opportunities for personal growth. Adolescents identified physical health, particularly sleep, physical activity, and daily routines, as an important contributor to wellbeing. While many recognized the value of healthy lifestyles, they also described difficulties maintaining these practices due to academic demands, time pressure, and competing responsibilities. These findings echo earlier research demonstrating strong associations between physical health and mental, emotional, and social wellbeing during adolescence (Bourke & Geldens, 2007a; Fattore et al., 2009; Thorburn, 2018; Marques et al., 2017; Hargreaves & Shirley, 2021; Avedissian & Alayan, 2021). The adolescents’ accounts underscore the importance of addressing contextual and structural barriers when promoting physical wellbeing.

Social relationships emerged as a central factor influencing adolescents’ wellbeing. Supportive family relationships, close friendships, and a sense of belonging were consistently described as sources of emotional security and acceptance. These findings are consistent with a large body of research highlighting social relationships as key predictors of adolescent wellbeing (Bourke & Geldens, 2007a; Awartani et al., 2008; Fattore et al., 2009; Easthope & White, 2006; Watson et al., 2012; Pulimeno et al., 2020). At the same time, adolescents described the evolving nature of friendships and the ambivalent role of social media. While digital platforms facilitated connection, they also introduced pressures related to comparison, appearance, and self-worth. This reflects mixed findings in existing research on social media and wellbeing, where both positive and negative associations have been identified (Bloemen & De Coninck, 2020).

Mental health was closely intertwined with adolescents’ experiences of wellbeing, and some participants used the two concepts interchangeably. Adolescents demonstrated awareness of strategies that could support mental health, such as mindfulness, self-care, therapy, and spending time in nature. However, many reported difficulties implementing these strategies consistently due to time constraints. Spending time in nature, particularly within the Norwegian cultural context, was described as beneficial, aligning with research highlighting positive associations between nature-based activities and wellbeing among young people (Roberts et al., 2020). Despite increased openness around mental health, stigma related to help-seeking remained a significant concern. Adolescents expressed reluctance to disclose mental health struggles within their social circles, which may limit access to support. Furthermore, many adolescents expressed a desire to develop resilience and coping skills, while also reporting uncertainty about how to translate these concepts into practice, a pattern also identified in earlier research (Fattore et al., 2009).

Academic performance pressure was identified as a significant source of stress, particularly in the later years of upper secondary education. Concerns about grades and future opportunities were closely linked to emotional wellbeing. These findings align with Norwegian research documenting academic pressure as a key challenge for adolescents, even in contexts with strong welfare systems (Bakken, 2019; Klomsten & Uthus, 2020). Importantly, adolescents emphasized the protective role of supportive school environments. Positive teacher–student relationships, a sense of safety, and feeling valued at school were described as mitigating academic stress and supporting wellbeing. These findings are consistent with research demonstrating the importance of school climate and teacher support for adolescent wellbeing (Eckersley et al., 2006; Watson et al., 2012; Ecclestone & Hayes, 2009; Pulimeno et al., 2020; Kiuru et al., 2020; Spilt et al., 2011; Milfont & Denny, 2018; Lin et al., 2022; Morin, 2020).

Personal growth emerged as a meaningful contributor to wellbeing. Adolescents associated wellbeing with self-awareness, autonomy, purpose, creativity, and the ability to manage everyday challenges. Engagement in hobbies and meaningful activities was described as supporting emotional balance and self-confidence. These findings resonate with earlier research linking personal development to positive wellbeing outcomes (Adams et al., 2000; J. White, 2011; Ekornes & Øye, 2022; Avedissian & Alayan, 2021). Adolescents expressed a clear preference for learning coping and life skills from trusted adults, such as teachers or other professionals, and through interactive and experiential approaches. Interest in technology-based supports further highlights the potential for developing accessible, youth-centered interventions (Vella-Brodrick et al., 2020; Vella-Brodrick et al., 2022).

The findings suggest that adolescent wellbeing is shaped by the interaction of individual experiences and broader social, educational, and cultural contexts. Schools emerge as key arenas for wellbeing promotion, not only through academic instruction but also through relational support and opportunities for skill development. Interventions that involve trusted adults, prioritize supportive relationships, and provide practical coping strategies may be particularly effective in strengthening adolescents’ wellbeing in Norway and comparable contexts.

## 5. Conclusions

This paper explores adolescents’ perceptions of wellbeing in the Norwegian context. The findings suggest that young people’s perceptions of wellbeing are multidimensional, include physical, mental, and social aspects, and emphasize the importance of personal growth in promoting wellbeing. The factors that hinder adolescents’ wellbeing, among others, are academic pressure, social media, and time constraints. The study has important implications for those working with young people to promote their health and development, particularly in educational settings. By taking a multidimensional approach to promoting wellbeing that includes addressing physical, emotional, and social aspects, educators, and practitioners can better support young people’s wellbeing. Additionally, by recognizing the importance of social relationships in promoting wellbeing, educators can create environments that foster positive relationships among young people. The study’s findings highlight the critical need for interventions that address adolescents’ longing to learn how to handle hard and demanding life situations. Adolescents expressed a strong desire to acquire coping skills, which could help them achieve personal growth, resilience, and improve their mental health. Further research is needed to develop and evaluate effective interventions that meet adolescents’ preferences and needs. While this study has some limitations, such as its relatively small sample size and the specific cultural context in which it was conducted, its findings provide a useful foundation for further research on young people’s perceptions of wellbeing.

Future research could explore how young people’s perceptions of wellbeing may differ across different cultural contexts or age groups, and how different interventions can support young people’s wellbeing. It is worthwhile to delve into the perspectives of adolescents regarding wellbeing in relation to policy approaches (Fattore et al., 2009). The responses about wellbeing received from young individuals in this research encompasses their ability to competently embrace the challenges that life presents, while also highlighting their concerns regarding friendship and establishing long-lasting relationships. These relationships are seen as crucial, as they involve fundamental elements such as love, care, kindness, trust, and respect. Moreover, their understanding of overall wellbeing revolves around various aspects, including school, home, family, friends, as well as enjoyment, engagement in activities, and personal development. The findings of this study suggest that wellbeing ought to be comprehensively understood and integrated into policies and practices as a holistic concept, rather than being fragmented and focusing solely on one aspect of wellbeing at a time. Furthermore, the findings emphasize that wellbeing should be an enduring part of the educational experience, ensuring its sustainability beyond a specific timeframe. To achieve this, a holistic approach involving multiple stakeholders is necessary, including parents, the community, teachers, school leadership, and other relevant parties. By fostering collaboration and collective responsibility, such an approach can effectively promote and support the overall wellbeing of adolescents within the educational context.

## Figures and Tables

**Table 1:** The main themes and sub-themes.

Main Themes	Sub Themes
The definition of wellbeing	Hard to define and understand Multidimensional Subjective and objective aspects Numerous determinants impacting wellbeing
Physical health	Healthy eating Regular exercise Adequate sleep Body image
Social relationships	Friendship Family support Romantic relationships Social media Trust, love, care, respect
Mental health	Stigmatization Longing to learn coping strategies Stress management strategies Navigating adversity with resilience and positivity Time management strategies
Academic performance pressure	School environment Academic achievement The role of social media Peer pressure Future outcomes
Personal growth	Self-awareness, positive sense of self, self-discipline, strategies to mitigate academic performance pressure. Purpose and meaning in life Autonomy (Mastery over everyday life) Creativity

## Data Availability

The data that support the findings of this study are available from the corresponding author upon reasonable request. Due to ethical and privacy restrictions, the data are not publicly available.
